# White Matter Hyperintensity Burden Is Associated With Hippocampal Subfield Volume in Stroke

**DOI:** 10.3389/fneur.2020.588883

**Published:** 2020-10-26

**Authors:** Mark R. Etherton, Panagiotis Fotiadis, Anne-Katrin Giese, Juan E. Iglesias, Ona Wu, Natalia S. Rost

**Affiliations:** ^1^Department of Neurology, J. Philip Kistler Stroke Research Center, Massachusetts General Hospital, Harvard Medical School, Boston, MA, United States; ^2^Department of Radiology, Martinos Center for Biomedical Imaging, Massachusetts General Hospital, Boston, MA, United States

**Keywords:** acute ischemic stroke, white matter disease, vascular dementia, MRI, hippocampus

## Abstract

White matter hyperintensities of presumed vascular origin (WMH) are a prevalent form of cerebral small-vessel disease and an important risk factor for post-stroke cognitive dysfunction. Despite this prevalence, it is not well understood how WMH contributes to post-stroke cognitive dysfunction. Preliminary findings suggest that increasing WMH volume is associated with total hippocampal volume in chronic stroke patients. The hippocampus, however, is a complex structure with distinct subfields that have varying roles in the function of the hippocampal circuitry and unique anatomical projections to different brain regions. For these reasons, an investigation into the relationship between WMH and hippocampal subfield volume may further delineate how WMH predispose to post-stroke cognitive dysfunction. In a prospective study of acute ischemic stroke patients with moderate/severe WMH burden, we assessed the relationship between quantitative WMH burden and hippocampal subfield volumes. Patients underwent a 3T MRI brain within 2–5 days of stroke onset. Total WMH volume was calculated in a semi-automated manner. Mean cortical thickness and hippocampal volumes were measured in the contralesional hemisphere. Total and subfield hippocampal volumes were measured using an automated, high-resolution, *ex vivo* computational atlas. Linear regression analyses were performed for predictors of total and subfield hippocampal volumes. Forty patients with acute ischemic stroke and moderate/severe white matter hyperintensity burden were included in this analysis. Median WMH volume was 9.0 cm^3^. Adjusting for intracranial volume and stroke laterality, age (β = −3.7, *P* < 0.001), hypertension (β = −44.7, *P* = 0.04), WMH volume (β = −0.89, *P* = 0.049), and mean cortical thickness (β = 286.2, *P* = 0.006) were associated with total hippocampal volume. In multivariable analysis, age (β = −3.3, *P* < 0.001) and cortical thickness (β = 205.2, *P* = 0.028) remained independently associated with total hippocampal volume. In linear regression for predictors of hippocampal subfield volume, increasing WMH volume was associated with decreased hippocampal-amygdala transition area volume (β = −0.04, *P* = 0.001). These finding suggest that in ischemic stroke patients, increased WMH burden is associated with selective hippocampal subfield degeneration in the hippocampal-amygdala transition area.

## Introduction

Post-stroke cognitive impairment/dysfunction (PSCID) is highly prevalent and associated with poor global outcomes (1–3). Recognition of the factors that predispose to PSCID is therefore clinically important, as early identification of vulnerable patients could guide individualized therapies focused on PSCID prevention and rehabilitation. Along these lines, identifying clinically relevant biomarkers that identify patients at risk for poor stroke outcomes is of major clinical interest (4). White matter hyperintensities of presumed vascular origin (WMH), as a marker of cerebral small-vessel disease, are a recognized risk factor for poor outcomes after ischemic stroke. Specifically, increased burden of WMH has been associated with worse functional outcomes after ischemic stroke (5), poor ischemic tissue outcomes (6), and PSCID (7–10). Regarding PSCID, total WMH burden is an independent determinant of poor cognitive outcomes after ischemic stroke (7, 11, 12). Extending beyond total WMH burden, additional studies have shown that the location of WMH is relevant with periventricular and deep WMH being associated with cognitive performance after stroke (13–16). Moreover, a lesion-symptom mapping-based approach identified the locations of WMH with additional impact on post-stroke cognitive beyond those effects from the acute infarct lesion (10). Finally, in a comparison of patients with minor stroke, the addition of other markers of small-vessel disease to WMH volume did not improve prediction accuracy of PSCID (9). While these observations exemplify the clinical relevance of WMH as a biomarker to identify patients at high risk for poor functional and cognitive outcomes after stroke, it remains unclear how increasing WMH burden predisposes to PSCID.

One potential hypothesis is that there is an interrelationship between cerebral small-vessel disease and neurodegenerative pathology that contributes to the development of PSCID (2, 5, 17). For example, in patients with ischemic stroke or transient ischemic attack, medial temporal lobe atrophy has been shown to be associated with qualitative measures of WMH and PSCID (18). In chronic stroke patients, WMH burden has also been associated with hippocampal atrophy and the late development of PSCID (17, 19). These observations of an association between WMH, as a marker of small-vessel disease, and total hippocampal volume, a radiographic marker associated with neurodegenerative diseases, suggest an interaction between small-vessel disease and neurodegenerative pathology that predisposes to PSCID. These studies, however, were largely in the chronic stages of stroke and focused on total hippocampal volume. In addition, the cytoarchitecture of the hippocampus is segregated whereby there are unique subfields that comprise different parts of the hippocampal circuitry and, based on their projections to different brain regions, have distinct roles for hippocampal function and memory (20, 21). For these reasons, an investigation into the relationship between WMH and the hippocampal subfields in acute ischemic stroke patients holds potential to further characterize the interaction between WMH and hippocampal atrophy and any differential effect on select hippocampal subfields. Secondarily, this investigation in the acute stage may elucidate another mechanism of how WMH predisposes to the development of PSCID.

The aim of this study was to gain further insight into the relationship between cerebral small vessel disease and hippocampal degeneration in an acute ischemic stroke population. We therefore performed an *in vivo* investigation of hippocampal subfield volumes and WMH volume. Our hypothesis was that because the hippocampus is a heterogeneous structure with distinct subfields with differential roles in learning and memory (22), WMH may be associated with degeneration of select hippocampal subfields.

## Materials and Methods

All aspects of this study and the use of human participants were approved by the local Institutional Review Board in October 2013. Written informed consent was obtained from all participating subjects or their surrogates.

### Subjects, Inclusion/Exclusion Criteria

**Figure 1:**
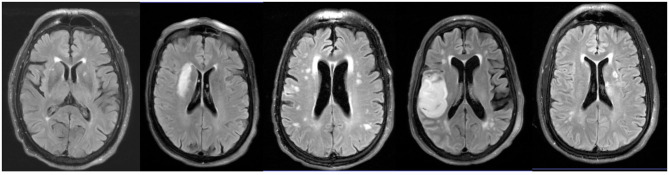
WMH location in AIS patients with moderate to severe WMH. Representative axial FLAIR images of WMH location in AIS patients enrolled in this study.

Consecutive subjects presenting to our institution and able to undergo a brain MRI within 24 h of stroke onset were screened for eligibility. All subjects with confirmed AIS on MRI that were able to receive gadolinium-based contrast and had radiographic evidence of moderate to severe WMH burden (Fazekas ≥ 2, Figure 1) were eligible for enrollment (23).

### Clinical Variables

Age, sex, race, prestroke functional status, and medical comorbidities were ascertained from the patient or their surrogate or otherwise from the medical record. Admission NIH stroke scale (NIHSS) was performed by a trained neurologist on clinical service at the time of patient presentation. Stroke subtype was determined according to the Trial of Org 10172 in Acute Stroke Treatment classification by a trained neurologist (24). The modified Rankin scale (mRS) was used to determine functional outcomes at 90 days post-stroke (25).

### Imaging Acquisition and Analysis

A research 3-T MRI was obtained 2–5 days post-stroke using a Siemens Skyra scanner. MP-RAGE sequences were acquired with the following parameters: TR/TE/TI = 2,530/1.69/1,100–1,300 ms, flip angle = 7°, 1 mm isotropic resolution. FLAIR sequences were acquired with the following parameters: TR/TE/TI = 9,000/119/2,200–2,500 ms, 5 mm slice thickness with 1 mm gap, and in-plane resolution of 0.86 × 0.86 mm^2^.

WMH lesions were outlined in both hemispheres in a semi-automated manner on the FLAIR sequences using MRIcro software as described previously (26). Briefly, masks for ipsi- and contralesional WMH were constructed in MRIcro (www.mricro.com) using the axial FLAIR sequence and applying a signal intensity threshold followed by manual editing.

Cortical reconstruction and volumetric segmentation were performed using FreeSurfer 6.0 image analysis suite (http://surfer.nmr.mgh.harvard.edu) as described previously (27). Briefly, this processing includes motion correction and averaging of multiple volumetric T1 weighted images, removal of non-brain tissue, automated Talairach transformation, segmentation of subcortical white matter and deep gray matter volumetric structures, tessellation of the gray matter white matter boundary, automated topology correction, and surface deformation following intensity gradients to optimally place the gray/white and gray/cerebrospinal fluid borders (28, 29). To minimize the influence of the acute stroke on the automated hippocampal segmentation, the hippocampal volumetric estimates and mean cortical thickness were acquired in the hemisphere contralateral to the acute infarct. The algorithm uses a generative framework and Bayesian inference in order to fit a high-resolution probabilistic atlas of the hippocampal subregions—derived from highly detailed manual delineations made on ultra-high resolution *ex vivo* MRI—to a target MRI scan (30). An advantage of the method is its adaptiveness to variations in MRI contrast and image intensity profiles, which is desirable when processing images with pathology, as in this study. For each subject, the T1-weighted structural image, surface models, and hippocampal segmentation were visually inspected for accuracy and manual corrections were performed for tissue misclassification when necessary. Estimated Total Intracranial Volume (eTIV) was calculated using FreeSurfer version 6.0.

### Statistical Analysis

No prestroke disability was defined as a mRS score of 0. Tobacco use was defined as active or any history of smoking. Excellent outcome was defined as mRS < 2 at 90 days post-stroke. The ipsilesional and contralesional WMH volumes were summed to obtain the total WMH volume (WMHv). The point-biserial correlation coefficient was determined for the correlation between WMHv and stroke laterality. Linear regression was performed to determine clinical and radiographic factors associated with hippocampal volume and cortical thickness adjusting for eTIV and stroke laterality (RStudio 1.0.153). For each dependent variable, all variables demonstrating a nominal *P*-value <0.05 in univariable analysis were incorporated into the multivariable backward stepwise linear regression model. A backward stepwise approach was pursued to identify a reduced model that best explains the data for each variable. For linear regression of hippocampal subfield volumes, eTIV and stroke laterality were again included as covariates to adjust for any effects of head size or laterality on the regression results. The Bonferroni correction was applied for multiple comparisons.

## Results

**Figure 2:**
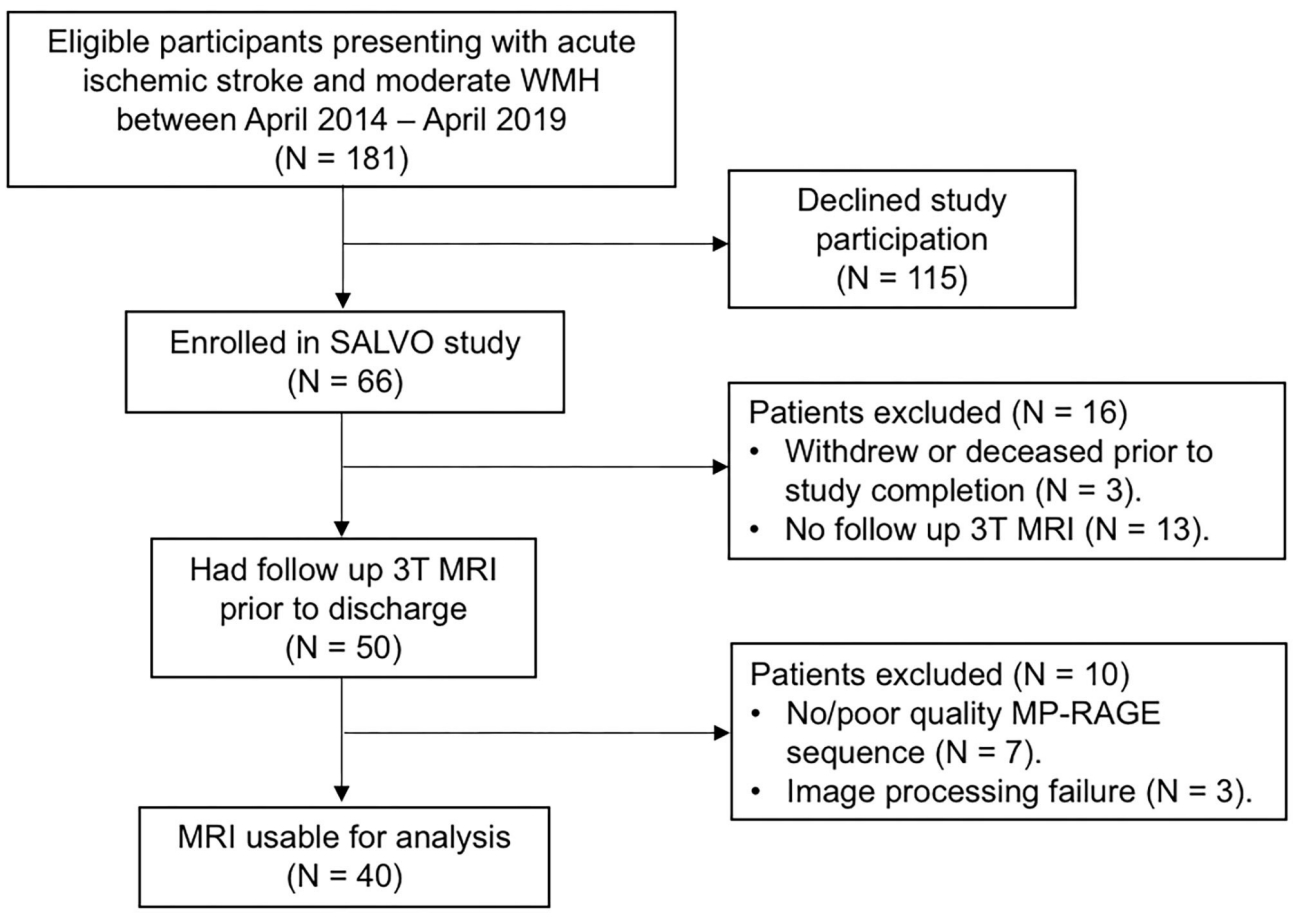
Study inclusion/exclusion criteria.

Forty subjects met inclusion criteria for this analysis (Figure 2; see Table 1 for details of baseline demographics). Median NIHSS was 4.5. Median WMH volume was 9.0 cm^3^ (Supplementary Figure 1). The median Fazekas scores were 2 (interquartile range; IQR 1, 3) for periventricular WMH and 1 (IQR 1, 2) for deep WMH. Strokes attributed to small-vessel occlusion represented 15% of the population. There was no significant correlation between WMHv and stroke laterality (*r* = −0.06, *P* = 0.70). In univariable linear regression for predictors of total hippocampal volume, increasing age, hypertension, and WMHv were associated with decreased hippocampal volume, while cortical thickness showed a positive association (Table 2). In multivariable, backward, stepwise linear regression, increasing age and cortical thickness were independent predictors of total hippocampal volume (Table 2).

**Table 1:** Clinical and radiographic characteristics of 40 AIS patients.

	**Values**
Age, *y*, mean (SD)	68.0 (10.6)
Male, *n* (%)	27 (67.5)
White race, *n* (%)	37 (92.5)
No prestroke disability, *n* (%)	36 (90.0)
**Past medical history**, ***n*** **(%)**	
Atrial fibrillation	10 (25.0)
Dementia	0 (0.0)
Diabetes mellitus	7 (17.5)
Hyperlipidemia	24 (60.0)
Hypertension	28 (70.0)
Prior ischemic stroke	3 (7.5)
Tobacco use	25 (62.5)
**Stroke subtype**, ***n*** **(%)**	
Large-artery atherosclerosis	8 (20.0)
Cardioembolism	14 (35.0)
Small-vessel occlusion	6 (15.0)
Stroke of undetermined etiology	12 (30.0)
**Clinical and radiographic data**	
NIHSS score, median (IQR)	4.5 (2.0, 11.0)
WMH volume, cm^3^, median (IQR)	9.0 (4.3, 17.8)
Left hemisphere stroke, *n* (%)	15 (37.5)
Hippocampal volume, mm^3^, median (IQR)	391.5 (367.3, 448.4)
Excellent outcome, *n* (%)	25 (62.5)

**Table 2:** Linear Regression for variables associated with total hippocampal volume.

	**Univariable**		**Multivariable**	
**Variable**	**Estimate**	***P*-value**	**Estimate**	***P*-value**
Age	−3.7	**0.0003**	−3.3	**0.0005**
Female sex	−22.6	0.42		
Atrial fibrillation	−25.2	0.32		
Diabetes mellitus	−14.4	0.62		
Hyperlipidemia	−12.4	0.56		
Hypertension	−44.7	**0.04**		
Prior ischemic stroke	−33.8	0.39		
No prestroke disability	12.1	0.73		
Tobacco use	0.37	0.99		
WMHv	−0.89	**0.049**		
Mean cortical thickness	286.2	**0.006**	205.2	**0.028**

*Multivariable initial linear regression model*.

Hippocampal volume ~β_0_ + β_1_^*^Age + β_2_^*^Hypertension + β_3_^*^WMHv + β_4_^*^Cortical thickness + β_5_^*^eTIV + β_6_^*^Stroke laterality.

^*^All variables with P values < 0.05 in the univariable analysis were input into the initial multivariable regression model.

*Multivariable final backward stepwise linear regression model*.

Hippocampal volume ~ β_0_ + β_1_^*^Age + β_2_^*^Cortical thickness + β_3_^*^eTIV.

*Bolded values indicate statistical significance of p < 0.05*.

Age (β = −0.003, *P* = 0.053) and hypertension (β = −0.08, *P* = 0.018) were associated with decreased mean cortical thickness in univariable analysis, while adjusting for eTIV and stroke laterality. In the multivariable model, however, only hypertension was a significant independent predictor of mean cortical thickness (β = −0.07, *P* = 0.043). WMHv was not associated with mean cortical thickness when adjusted for eTIV and stroke laterality (β = −0.0001, *P* = 0.90).

**Figure 3:**
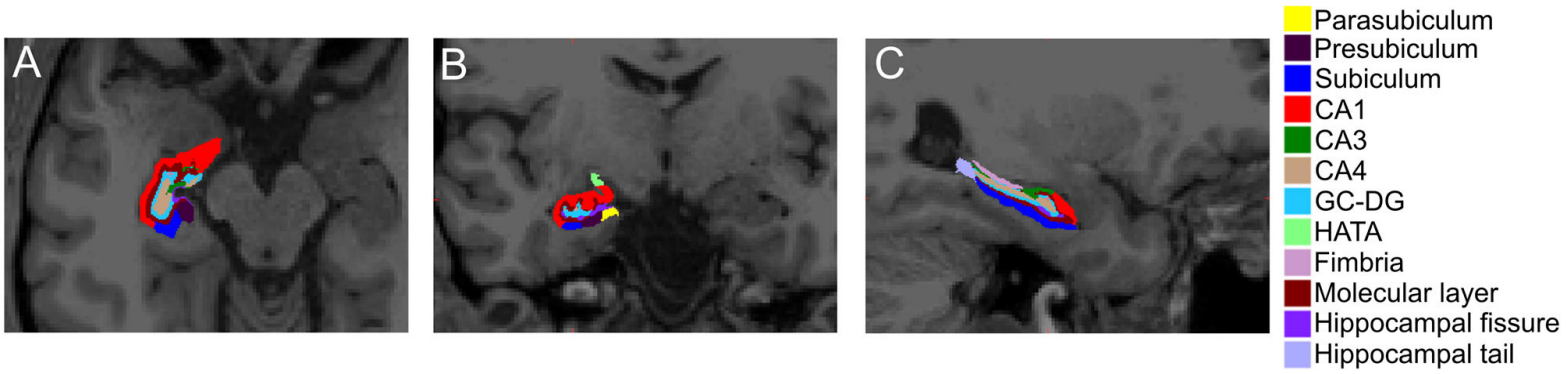
Hippocampal subfield segmentation in the hemisphere contralateral to the acute stroke. Representative image of hippocampal subfield automated segmentations in the **(A)** axial, **(B)** coronal, and **(C)** sagittal orientations. CA, cornu ammonis; GC-DG, granule cell layer of dentate gyrus; HATA, hippocampal-amygdala transition area.

Hippocampal subfield volumes were subsequently segmented (Figure 3). Linear regression for predictors of hippocampal subfield volume demonstrated that WMHv was an independent predictor of Hippocampal Amygdala Transition Area (HATA) volume (Table 3). When a clinical diagnosis of hypertension was included in the regression model, WMHv remained an independent predictor of HATA volume (β = −0.03, *P* = 0.003).

**Table 3:** Linear regression for association of WMHv with hippocampal subfield volume.

**Hippocampal subfield**	**Estimate**	***P* value**
Parasubiculum	−0.01	0.32
Presubiculum	−0.10	0.087
Subiculum	−0.13	0.078
CA1	−0.12	0.18
CA3	−0.03	0.34
CA4	−0.04	0.24
GC-DG	−0.06	0.12
Molecular layer	−0.15	0.064
Fimbria	−0.05	0.15
Hippocampal tail	−0.18	0.006
Hippocampal fissure	−0.0005	0.98
HATA	−0.04	**0.001**

*CA, cornu ammonis; GC-DG, granule cell layer of dentate gyrus; HATA, hippocampal-amygdala transition area*.

*Multivariable linear regression model*.

Hippocampal subfield volume ~ β_0_ + β_1_^*^ WMHv + β_2_^*^eTIV + β_3_^*^Stroke laterality. Bolded value denotes statistically significant after correcting for multiple comparisons.

Lastly, we pursued subgroup analysis of the association between WMHv and HATA volume based on stroke laterality. In right hemisphere stroke patients (*N* = 25), increased WMHv was significantly associated with reduced left HATA volume (β = −0.03, *P* = 0.008) when adjusting for eTIV. In patients with left hemisphere stroke (*N* = 15), increased WMHv was associated with decreased right HATA volume (β = −0.03, *P* = 0.19), but did not achieve statistical significance, when adjusting for eTIV.

## Discussion

In a cohort of AIS patients with moderate to severe WMH burden and no prestroke dementia, we show that WMHv is a negative predictor of total hippocampal volume and selectively associated with volume of the HATA subfield. We also show that a clinical diagnosis of hypertension and increasing age are associated with decreased hippocampal volume and mean cortical thickness. There are several important implications of these findings with regards to how WMH and small-vessel disease risk factors (e.g., hypertension) may predispose to PSCID. In addition, these findings provide potential insight into an *in vivo* relationship between markers of cerebral small vessel and neurodegenerative diseases.

WMH burden has previously been shown to be associated with PSCID (9) and hippocampal atrophy in healthy, community-dwelling adults (31), patients with mild cognitive impairment/Alzheimer's dementia (32, 33), or stroke (18, 34). Analysis of 511 nondemented elderly subjects in the Rotterdam study, a prospective study of community-dwelling adults, showed that qualitative measures of increased WMH were associated with atrophy of the hippocampus and amygdala (31). In patients with stroke, one analysis of individual patient level data from the Virtual International Stroke Trials Archive found that, in a population of patients with predominantly small-vessel occlusive mediated ischemic stroke, markers of small-vessel disease were associated with medial temporal lobe atrophy and PSCID (18). Another retrospective cohort study of patients admitted to a hospital stroke service demonstrated that medial temporal lobe atrophy was associated with verbal memory skills. In another study of chronic stroke patients, medial temporal lobe atrophy correlated with WMH burden and PSCID (19). Based on the role of the hippocampus in cognition, these findings suggest that increasing WMH burden predisposes to PSCID, at least in part, through its association with hippocampal atrophy. Because the hippocampus receives input from the prefrontal and parietal-temporal association areas, one hypothesis is that white matter injury to these projection tracts reduces hippocampal input causing secondary hippocampal atrophy and resultant poor cognitive outcomes.

In support of this hypothesis, longitudinal analysis of 503 nondemented subjects with small-vessel disease, as part of the RUN DMC study, demonstrated that the interaction term of WMH and hippocampal atrophy significantly improved model accuracy for both working and episodic memory (35). Moreover, increased WMH burden was associated with decline in episodic memory and this association was not causally mediated by hippocampal atrophy in mediation analysis (35). These findings suggest that the interaction of small-vessel disease and neurodegenerative pathology may drive PSCID.

We also show the novel finding that increasing WMH burden is selectively associated with decreased volume of the HATA subfield of the hippocampus. Subgroup analysis for stroke laterality, showed that increased volume of the left HATA (e.g., patients with right hemisphere stroke) but not right HATA was associated with decreased WMH burden. Of note, both hemispheres showed an association between increasing HATA volume and decreased WMH burden, however, the lack of statistical significance with the right HATA is likely due to the smaller sample size of left hemisphere stroke patients in this study (*N* = 15). The association between HATA volume and WMH burden is of clinical interest given the potential to provide insight into the mechanism of how increased chronic white matter structural injury is associated with PSCID and poor functional outcomes (5, 14, 36). Based on the connectivity of the HATA, with projections to the medial amygdala (37), and secondarily the many projections to the amygdala from other brain regions, such as the prefrontal cortex and thalamus, it seems plausible that WMH could manifest clinically by the disruption of critical white matter tracts to hippocampal subfields. Along these lines, abnormalities in hippocampal subfield volumes have been reported in other diseases including bipolar disorder (38), depression (39), and neurodegenerative disorders (40, 41). Specific to the HATA, studies in healthy adults and patients with neurologic disorders have suggested a relationship with cognitive function. One study of 275 healthy adults demonstrated that both immediate and delayed recall scores were associated with HATA volume (42). Furthermore, in patients with Parkinson's disease, HATA volume has been shown to be predictive of the conversion to mild cognitive impairment (43). As the HATA is a key component of the limbic system that plays a critical role in emotional learning and memory and social cognition (44), these findings offer some insight into the mechanisms of how increased WMH burden contributes to PSCID.

We also demonstrate that the application of an ultra-high-resolution computational atlas for quantitative segmentation of hippocampal volumes is feasible in ischemic stroke patients contemporaneous with the index stroke. Hippocampal subfield segmentation performed using an *ex vivo* ultra-high-resolution atlas, such as we employed here, has been shown to provide a more accurate estimate of hippocampal volume than *in vivo* atlases or whole hippocampal segmentations (30). As such, incorporation of this approach into post-stroke clinical assessments may have utility in identifying stroke patients particularly vulnerable PSCID development.

There are several limitations of our study that are important to consider when interpreting the overall generalizability including. First, this was a study of a relatively small sample of AIS patients with moderate to severe WMH burden. We would maintain, however, that the inclusion criteria of this study, enriches the cohort for individuals with advanced small-vessel disease that are most at risk for development of PSCID. A second limitation is the absence of specific assessments of cognitive outcomes, which would afford an analysis of hippocampal subfield volumes and post-stroke cognitive function and further inform on the clinical relevance of these findings. Future studies therefore should assess the relationship between white matter structural injury, hippocampal subfield volume, and long-term cognitive outcomes post-stroke. Regarding the neuroimaging approach, while we showed feasibility of performing research grade MRIs in the late acute to subacute phase of ischemic stroke, there is potential bias from the spatial resolution of 3T MRI for hippocampal volume analysis and errors in automated hippocampal volume extraction. We would assert, however, that: (i) the HATA is one of the subregions that the *ex vivo* atlas extracts most accurately (30), since it shows strong contrast with neighboring CSF (this is, e.g., in contrast with internal subregions such as CA4); (ii) that the similar relationship between WMH burden and hippocampal size in stroke-free (32, 33) and chronic stroke individuals (19) corroborates our findings; and (iii) that the automated hippocampal volume extraction, with visual inspection of segmentation for obvious errors, minimizes inter-rater variability and is therefore a strength of this approach. Clearly, additional studies are needed investigating hippocampal volume at the time of index stroke in relation to longitudinal cognitive assessments.

## Conclusions

Increasing WMH burden is selectively associated with decreased HATA volume in AIS patients. The selective association of WMHv, as a marker of cerebral small vessel disease, and the hippocampal subfield volume of the HATA may inform on the *in vivo* mechanisms of PSCID.

## Data Availability Statement

The raw data supporting the conclusions of this article will be made available upon reasonable request to the authors and pending approval of the local Institutional Review Board.

## Ethics Statement

The studies involving human participants were reviewed and approved by Massachusetts General Hospital Institutional Review Board. The patients/participants provided their written informed consent to participate in this study.

## Author Contributions

ME designed and conceptualized study, analyzed the data, data acquisition and interpretation, and drafted the manuscript for intellectual content. PF and A-KG performed data acquisition and revised manuscript for intellectual content. JI interpreted the data and revised the manuscript for intellectual content. OW and NR designed and conceptualized the study, analyzed the data, and revised manuscript for intellectual content. All authors contributed to the article and approved the submitted version.

## Conflict of Interest

The authors declare that the research was conducted in the absence of any commercial or financial relationships that could be construed as a potential conflict of interest.
