# Supplementary material for: White Matter Hyperintensity Burden Is Associated With Hippocampal Subfield Volume in Stroke
Source: Front Neurol. 2020 Oct 26;11:588883. doi: 10.3389/fneur.2020.588883 (PMC7649326; doi:10.3389/fneur.2020.588883)
Supplement: Supplementary file 1 [file Data_Sheet_1.DOCX]

**Supplementary Figure 1: Volumetric measurements for A) WMH, B) mean hippocampal volume, and C) mean cortical thickness.**

**A**

**
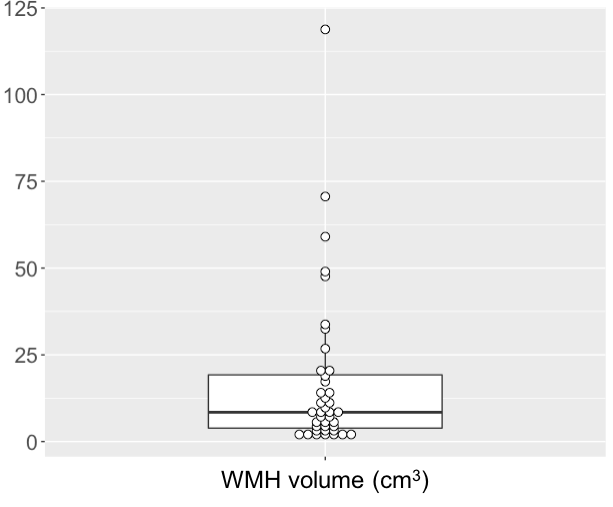
**

**B**

**
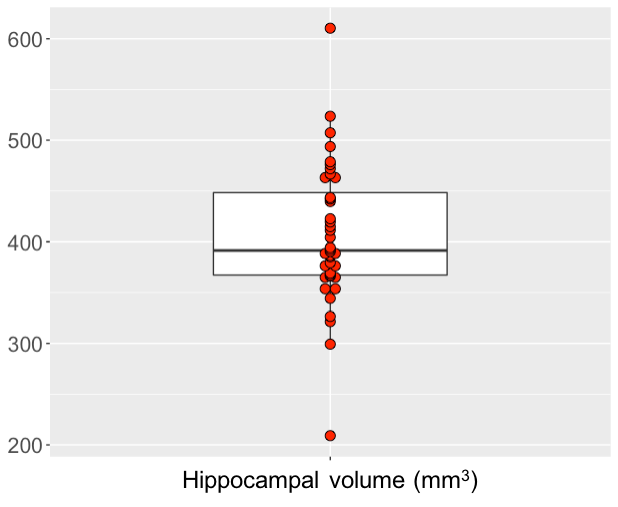
**

**C**

**
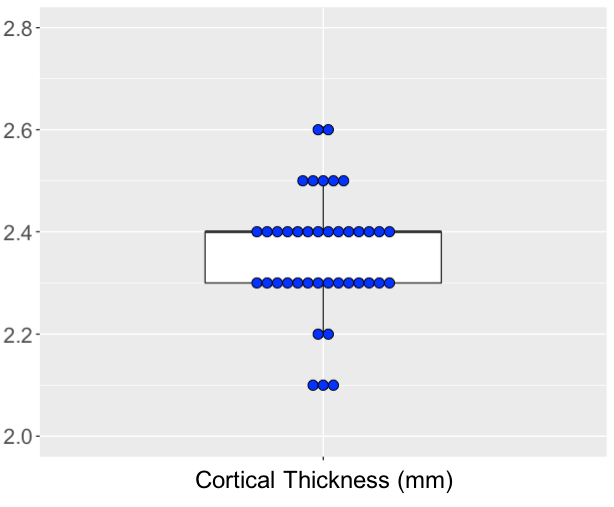
**
